# Editorial: Incretin-based therapies in the treatment of metabolic syndrome: expanding roles beyond weight management

**DOI:** 10.3389/fendo.2025.1676718

**Published:** 2025-08-15

**Authors:** Kexin Zhang, Xiaodong Sun, Lixin Li

**Affiliations:** ^1^ Department of Endocrinology and Metabolism, Shandong Provincial Key Medical and Health Laboratory of Endocrinology and Metabolic Diseases, Affiliated Hospital of Shandong Second Medical University, Weifang, China; ^2^ Clinical Research Center, Affiliated Hospital of Shandong Second Medical University, Weifang, China; ^3^ College of Health Professions, Central Michigan University, Mount Pleasant, MI, United States

**Keywords:** incretin, GLP-1, body weight, metabolic syndrome, GIP

Metabolic syndrome (MetS), characterized by a cluster of metabolic abnormalities such as obesity, dyslipidemia, and hyperglycemia, has become a major global public health concern (1). Incretin-based therapies, including glucagon-like peptide-1 (GLP-1) receptor agonists, dual GLP-1/glucose-dependent insulinotropic polypeptide (GIP) receptor agonists, and triple GIP/GLP-1/-glucagon receptor agonist, have been show to enhance energy expenditure, activate brown adipose tissue, and delay gastric emptying (4–6), demonstrating promising potential beyond glycemic control and weight loss (2, 3). Furthermore, novel dual and triple agonists further offer potential advantages through simultaneous targeting multiple pathways involved in energy expenditure. Growing evidence supports their broader metabolic and cardiovascular benefits, thus redefining their therapeutic role in MetS management. However, the mechanistic pathways and long-term safety of incretin-based therapy require further elucidation This Research Topic features three original research articles and one meta-analysis that collectively highlight the evolving landscape of incretin-based therapies in MetS.

The efficacy of incretin-based therapies in weight loss and glycemic control is well established. In a meta-analysis of eleven randomized controlled trials, Tian et al. evaluated the efficacy and safety of tirzepatide, a dual GLP-1/GIP receptor agonist, focusing on its effectiveness for weight loss and its dose-dependent effects in individuals with obesity or type 2 diabetes. Their findings confirmed tirzepatide’s exceptional efficacy in promoting both weight loss and glycemic improvement. Non-diabetic individuals experienced weight reductions of up to 17.86 kg, whereas patients with type 2 diabetes exhibited marked improvements in HbA1c. Tirzepatide also increased the proportion of patients achieving clinically meaningful weight loss compared with placebo. However, gastrointestinal side effects such as nausea, vomiting, constipation, especially at higher doses, may impact long-term adherence. While these effects are generally manageable and not associated with increased serious adverse events, the lack of long-term safety data calls for caution. Overall, tirzepatide represents a promising therapeutic option for metabolic disorders with a strong dose-response relationship Gastrointestinal side effects remain a concern. Its long-term success will depend on addressing practical challenges in patient care.

Pharmacogenomic studies and Genome−Wide Association Study (GWAS) have shown that genetic polymorphisms can significantly influence therapeutic responses (7). Building on this, Tonin et al. offer important insights into the genetic basis of differential responses to GLP-1RAs. Their study identified a significant association between the GLP1 receptor rs6923761 polymorphism and glycemic response, with G allele carriers showing greater reductions in HbA1c. These findings highlight the potential of genetic markers to guide personalized treatment strategies by identifying likely responders and non-responders, thereby reducing trial-and-error prescribing. This reinforces the clinical value of genetic testing in optimizing drug efficacy.

With the increasing prevalence of metabolic surgery (MS), weight regain and recurrent obesity have become common, highlighting the need for adjunctive therapies (8). Ouyang et al. investigated the impact of MS history on liraglutide efficacy and found that patients with prior MS and a baseline BMI ≥30.5 kg/m² experienced significantly less weight loss compared to non-surgical controls (≥5% total weight loss: 5.2% vs. 9.6%). These findings suggest possible GLP-1 receptor desensitization or downstream signaling alterations due to post-surgical metabolic remodeling that affect enteroendocrine cell distribution, highlighting the importance of considering surgical history in post-operative pharmacotherapy. Higher liraglutide doses (e.g., 3.0 mg/day) or next-generation dual or triple agonists such as tirzepatide or retatrutide may offer improved efficacy in this population. As bariatric procedures become more widespread, a better understanding of how anatomical and physiological changes impact drug response is critical for optimizing long-term treatment strategies. The study also underscores the need for individualized approaches to managing obesity in post-surgical patients.

Finally, optimizing incretin therapy requires identifying reliable predictors of treatment response. Song et al. investigate clinical predictors of response to liraglutide in patients with type 2 diabetes and MetS, aiming to refine patient selection. Their prospective study included 206 patients identified higher baseline HbA1c (>9.24%), elevated BMI (>26.89 kg/m²), and shorter diabetes duration (<7.99 years) as key predictors of favorable glycemic and weight-loss outcomes after six months of liraglutide therapy. ROC analysis yielded an AUC of 0.851, supporting the robustness of these indicators. Interestingly, nearly half of the cohort were non-responders, potentially due to the relatively low liraglutide dose (1.2 mg/day) used. This observation raises the possibility that dose escalation or early intervention in poorly controlled patients could improve outcomes. Their findings also emphasize the importance of individualized therapeutic strategies in optimizing the efficacy of incretin-based treatments. The authors also note the influence of unmeasured factors such as lifestyle or genetics, reinforcing the need for integrative predictive models.

In summary, incretin-based therapies offer promising benefits for MetS. This Research Topic highlights the safety and enhanced efficacy of dual agonists, the impact of genetic and clinical predictors on treatment response, the influence of MetS history on liraglutide efficacy, and the need for individualized treatment approaches. Advancing precision medicine and addressing long-term safety will be essential to optimize their use in MetS management.
